# Advances in human induced pluripotent stem cell (hiPSC)-based disease modelling in cardiogenetics

**DOI:** 10.1515/medgen-2025-2009

**Published:** 2025-04-08

**Authors:** Timon Seeger, Sandra Hoffmann

**Affiliations:** University Hospital Heidelberg Institute of Human Genetics Heidelberg Germany

**Keywords:** hiPSCs, cardiogenetics, disease modelling, genome editing, cardiac tissue engineering

## Abstract

Human induced pluripotent stem cell (hiPSC)-based disease modelling has significantly advanced the field of cardiogenetics, providing a precise, patient-specific platform for studying genetic causes of heart diseases. Coupled with genome editing technologies such as CRISPR/Cas, hiPSC-based models not only allow the creation of isogenic lines to study mutation-specific cardiac phenotypes, but also enable the targeted modulation of gene expression to explore the effects of genetic and epigenetic deficits at the cellular and molecular level.

hiPSC-based models of heart disease range from two-dimensional cultures of hiPSC-derived cardiovascular cell types, such as various cardiomyocyte subtypes, endothelial cells, pericytes, vascular smooth muscle cells, cardiac fibroblasts, immune cells, etc., to cardiac tissue cultures including organoids, microtissues, engineered heart tissues, and microphysiological systems. These models are further enhanced by multi-omics approaches, integrating genomic, transcriptomic, epigenomic, proteomic, and metabolomic data to provide a comprehensive view of disease mechanisms.

In particular, advances in cardiovascular tissue engineering enable the development of more physiologically relevant systems that recapitulate native heart architecture and function, allowing for more accurate modelling of cardiac disease, drug screening, and toxicity testing, with the overall goal of personalised medical approaches, where therapies can be tailored to individual genetic profiles.

Despite significant progress, challenges remain in the maturation of hiPSC-derived cardiomyocytes and the complexity of reproducing adult heart conditions. Here, we provide a concise update on the most advanced methods of hiPSC-based disease modelling in cardiogenetics, with a focus on genome editing and cardiac tissue engineering.

## Introduction

Inherited cardiovascular diseases such as cardiomyopathies as well as vascular and lipid disorders are significant causes of morbidity and mortality worldwide [1]. Understanding the genetic basis of these conditions is crucial for diagnosis, effective treatment and prevention of disease progression. Traditional animal models have played a key role in validating genetic cardiac conditions and in testing drug safety and efficacy in pre-clinical trials. However, due to significant differences between animal and human physiology, only 10–20 % of preclinically validated compounds are successful in clinical studies and gain approval as novel therapeutic approaches [2], highlighting the limitations of such models in recapitulating the genetic and phenotypic nuances of human heart diseases. Consequently, advanced human heart models are needed that are ideally patient-specific, reflecting the individual genetic background while closely mimicking the tissue environment under both physiological and pathological conditions.

More than 25 years ago, the first pluripotent stem cells were derived from preimplantation embryos as human embryonic stem cells (hESCs) [3], which enabled the generation of various hESCs-derived cell types in the years that followed. Thus, the availability of stem cell-derived human cardiomyocytes [4] has enabled the development of advanced models and has since revolutionised the field. In particular, the somatic reprogramming technology into human induced pluripotent stem cells (hiPSCs) [5] has facilitated the generation of virtually unlimited patient-derived cardiovascular cell types [6]. Advances in gene editing technologies such as CRISPR/Cas-based approaches have further facilitated precise genetic modifications [7–9]. Together, these innovations marked the beginning of a new era of in vitro cardiac disease modelling. This review explores the current landscape of hiPSC-based research in cardiogenetics, highlighting the integration of genome editing technologies and progress in cardiac tissue engineering approaches.

## Generation of hiPSC-derived cardiovascular cell types

**Figure 1:**
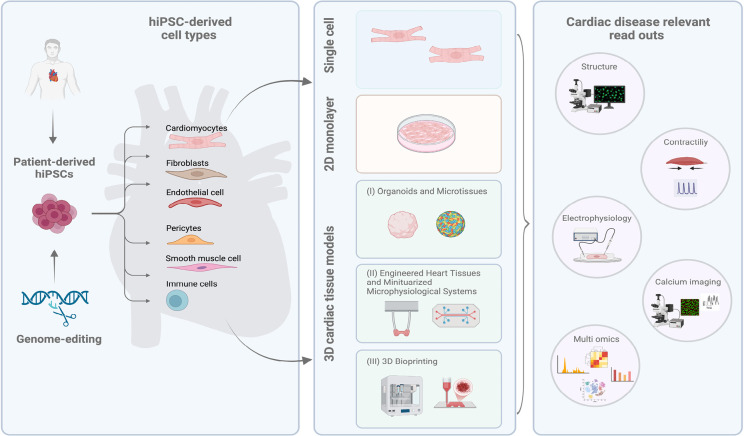
Human induced pluripotent stem cell (hiPSC)-derived cardiac disease modelling. Overview of hiPSC-derived cell types, including cardiomyocytes, fibroblasts, endothelial cells, pericytes, smooth muscle cells, immune cells (**left panel**). hiPSC-derived cardiac disease models encompassing single-cell approaches, two-dimensional (2D) monolayer cell culture models, and advanced three-dimensional (3D) cardiac tissue engineering based on three main strategies: (I) self-assembly of cells and extracellular matrix in organoid and microtissue cultures, (II) specialised devices direct cardiac tissue formation in engineered heart tissues and microphysiological systems, and (III) 3D bioprinting (**middle panel**). Summary of cardiac disease relevant read outs including structural integrity, cardiac contractility, electrophysiology, calcium imaging and multi-omics approaches (**right panel**). Created in BioRender.

The number of protocols to generate hiPSC-derived cardiomyocytes (hiPSC-CMs) in adherent two-dimensional (2D) monolayer [10–12] and three-dimensional (3D) suspension formats [13, 14] has increased exponentially over the past decade. However, despite the initial enthusiasm, it soon became evident that differentiated hiPSC-CMs rather resemble fetal cells and do not fully recapitulate the morphological, transcriptional, physical, electrical, and functional characteristics of adult cardiomyocytes [10, 15, 16]. They are small and round-shaped, with disorganised sarcomeres, sparse myofibrils, absent T-tubules, and underdeveloped intercalated discs, leading to weaker contractions and less efficient coupling compared to their adult counterparts [15]. Electrophysiologically, immature hiPSC-CMs show spontaneous beating characteristics, slower action potential propagation, unstable pacemaker activity, and limited calcium signalling, in contrast to adult cardiomyocytes, which exhibit faster conduction and efficient calcium handling due to mature ion channel expression. Additionally, immature hiPSC-CMs display a fetal-like expression profile with higher levels of fetal contractile protein isoforms [16]. The metabolic phenotype also differs, with hiPSC-CMs primarily utilising glycolysis for energy production, with lower mitochondrial density, immature structures and reduced oxidative phosphorylation activity, whereas adult cardiomyocytes predominantly rely on fatty acid oxidation for higher ATP production, supported by high mitochondrial density and efficiency [17]. Thus, substantial efforts have been made to improve this powerful model system. Extensive work has focused on promoting the maturation of hiPSC-CMs through optimised culture conditions, mechanical stimuli, and biochemical cues (as reviewed elsewhere, e.g. Ottaviani et al. [18]). Furthermore, considerable progress has been made in differentiating hiPSCs into various cardiomyocyte subtypes with unique functional properties, e.g. ventricular, atrial and nodal cardiomyocytes, to more accurately recapitulate the subtype-specific characteristics of cardiovascular diseases [19–21]. In this context, quality control of hiPSC-CMs is of critical importance and includes the validation of cardiac subtypes at the molecular level, the assessment of electrophysiological properties to distinguish action potential shapes, and the assessment of purity and maturity. Besides pathogenic mechanisms originating in cardiomyocytes, cardiovascular diseases may also arise from or be predominantly driven by non-cardiomyocyte cell types. A number of differentiation protocols have been established to derive various cell types from hiPSCs, including endothelial cells, vascular smooth muscle cells [22], pericytes [23], cardiac fibroblasts [24], and immune cells [25] (Fig. 1). Similarly, these hiPSC-derived cell types, also undergo rigorous phenotypic validation for marker expression, functional assays, and purity assessment. This allows for more accurate in vitro modelling of the complex cellular environment within the heart. Nevertheless, similar maturation challenges observed in cardiomyocytes also apply to other hiPSC-derived cardiovascular cell types, such as vascular cells, including endothelial and smooth muscle cells [26, 27] and cardiac fibroblasts [28]. These cells frequently exhibit fetal characteristics in structure, function, and gene expression, limiting their ability to fully replicate adult phenotypes. Continued refinement of these protocols is essential for generating more functional and homogeneous populations of these cells for clinical and research applications. Nevertheless, the models outlined have already contributed to a better understanding of genetic cardiovascular diseases, which will be discussed in more detail in the next section.

## Modelling inherited cardiovascular diseases using hiPSCs

Disease models based on hiPSCs offer a patient-specific platform to investigate the genetic causes of heart diseases [29]. By reprogramming somatic cells from patients with known genetic mutations or inherited cardiac conditions into hiPSCs, cardiovascular cell types can be generated that replicate the phenotypic characteristics of the underlying heart disease in vitro. This personalised approach is particularly valuable for uncovering the unique cellular and molecular mechanisms associated with various inherited cardiac conditions.

Hypertrophic cardiomyopathy (HCM) is mainly driven by mutations in genes encoding sarcomeric proteins, e.g. myosin heavy chain (*MYH7*) and myosin binding protein C3 (*MYBPC3*)*.* In rare cases, however, HCM can result from pathogenic variants in non-sarcomeric genes, while certain conditions, known as HCM phenocopies, can mimic its phenotype, such as lysosomal and glycogen storage disorders, cardiac amyloidosis, PRKAG2 cardiomyopathy, RASopathies (e.g., Noonan syndrome), mitochondrial diseases, and other metabolic disorders [30, 31]. It is characterised by pathological hypertrophy of the left ventricle, which causes outflow tract obstruction and stiffening of the heart muscle. This leads to diastolic heart failure and increases the risk of malignant arrhythmias, which can result in sudden cardiac death. Phenotypic aspects have been shown to be recapitulated in vitro in hiPSC-CMs [32, 33], allowing for further molecular investigation of the pathophysiological mechanisms driving the disease. On the contrary, dilated cardiomyopathy (DCM) clinically manifests with systolic heart failure due to impaired contractile functions as well as dilation of the left ventricle and thinning of its wall. In addition, malignant arrhythmias as well as sudden cardiac death occur regularly. The underlying causes of DCM are highly diverse; however, with the increasing application of deep sequencing technologies, the proportion of DCM cases attributed to genetic mutations is rising significantly. Pathogenic mutations have been identified in sarcomeric genes such as troponin T (*TNNT2*), myosin heavy chain 7 (*MYH7*), and titin (*TTN*). Additionally, mutations in genes associated with various cellular processes and structures have been implicated in DCM. For example, mutations in lamin A/C (*LMNA*) affect the nuclear envelope, while mutations in RNA-binding motif 20 (*RBM20*) disrupt post-transcriptional splicing, particularly of sarcomeric genes and other genes essential for myocardial function and calcium handling. Recent studies using hiPSC-derived models from DCM patients have revealed disease-relevant pathomechanisms, providing new insights and potential targets for innovative therapeutic approaches [34–38]. Beyond these most prominent hereditary cardiomyopathies, arrhythmogenic cardiomyopathies and ventricular tachycardias, associated with mutations in genes encoding desmosomal proteins, ion channels such as *SCN5A* [39, 40], and calcium-handling genes [41], have been effectively modeled using hiPSC-CMs. Similarly, long QT syndrome, caused by mutations in ion channel genes such as *KCNQ1* and *KCNH2*, has been recapitulated in these cells [42, 43]. Several studies successfully utilised hiPSC-based models to investigate the pathogenesis of cardiomyopathies linked to metabolic disorders and impaired mitochondrial bioenergetics, often attributed to genetic mutations in mitochondrial genes [44–46].

Beside modelling clear pathogenic mutations, hiPSC-CMs have emerged as a promising tool for already assessing the pathogenicity of variants of unknown significance [47]. In addition, first reports have evaluated patient-specific drug screening and toxicity testing employing hiPSC-CMs, taking advantage of the genetic background shared between the patient and the hiPSC line [48, 49]. Targeting specific mutations or genetic backgrounds allows for tailored approaches that optimise treatment for individual patients and may improve outcomes. However, to ensure broader applicability and facilitate the development of therapies for larger cohorts with similar syndromes but different mutations, it is crucial to identify common pathways or therapeutic targets shared across genetic variations within a syndrome, enabling both personalised and widely applicable treatments.

Taken together, hiPSC-based disease modelling of hereditary cardiomyopathies and genetically predisposed cardiovascular conditions still offers a tremendous potential for uncovering disease relevant molecular mechanisms leading to novel therapeutic approaches. However, despite the success of these models, significant limitations persist in the use of hiPSC-based model systems. hiPSC-CMs often exhibit structural and functional immaturity, which hampers their ability to fully mimic adult cardiovascular pathology. Furthermore, hiPSC-based research is laborious and complex, and prone to large variability between hiPSC-lines, protocols and laboratories. Finally, even in multicellular 3D models, the reductionist nature of in vitro systems – lacking the full physiological environment of the cardiovascular system – remains a major constraint. Consequently, significant research efforts have been, and continue to be, dedicated to advancing cardiac tissue engineering – an important aspect that will be discussed in more detail in a later section.

## CRISPR-based genome editing

An essential milestone in genetic disease modelling using hiPSCs was the integration of genome editing technologies, particularly the CRISPR/Cas9-based approach. The combined application of these two scalable approaches allows for precise manipulation of genetic variants, enabling the generation of isogenic hiPSC lines that differ solely in the mutation of interest. Additionally, fine-tuning of gene expression regulation can be achieved, providing deeper insights into the molecular mechanisms underlying biological functions and disease processes.

The CRISPR/Cas9 gene editing system, originally derived from the immune defense strategies of bacteria – specifically, *Streptococcus pyogenes* for the Cas9 enzyme – was successfully adapted for the use in mammalian cells about a decade ago [7–9]. CRISPR/Cas9 has become the most widely used genome editing tool due to its ability to target and modify any genomic sequence with remarkable precision and superior editing efficiency, along with its relative ease of use compared to other genome editing technologies. [50]. The system operates by utilising a guide RNA (gRNA) to direct the Cas9 enzyme to a particular DNA sequence where it can introduce double-strand breaks, which in turn leads to the activation of DNA-repair mechanisms: non-homologous end joining (NHEJ) and homology directed repair (HDR). The error-prone NHEJ pathway introduces insertions or deletions, resulting in frameshift mutations and is often employed to receive a complete gene knockout. In contrast, the HDR pathway uses a homology template (e.g. co-applicated single stranded DNA) for precise correction of introduced double-strand breaks with the desired sequence, making it suitable for introducing/correcting specific mutations or to insert transgenes like fluorescent proteins. Yet, many human cell types, including hiPSCs, exhibit relatively low efficiency in executing HDR. HDR efficiency is generally low in terminally differentiated cardiovascular cells, such as cardiomyocytes, due to their largely post-mitotic state and limited proliferation capacity, which restrict HDR activity to the S and G2 phases of the cell cycle. Strategies to overcome these limitations include inhibiting the competitive NHEJ pathway with chemical inhibitors and CRISPR modifications, manipulating the cell cycle, optimising the delivery and design of repair templates, stochastic enrichment of precise edits and enhancing the HDR machinery with exogenous proteins or small molecules [51–53]. As a major limitation, despite their highly locus specific way of action, off-target effects can occur based on DNA binding with mismatches.

Importantly, when the canonical NHEJ repair mechanism fails or is unavailable, alternative pathways such as microhomology-mediated end joining (MMEJ) and single-strand annealing (SSA) can facilitate the repair of DNA double-strand breaks. However, the prevalence and efficiency of these mechanisms may vary depending on factors such as cell type, cell cycle phase, and genomic context [54].

Alternative genome editing techniques have been developed, utilising catalytically inactive Cas9 proteins that retain the ability to target specific genomic locations, even when their ability to cut DNA is partially (nickase Cas9, nCas9) or entirely (dead Cas9, dCas9) disabled. The power of these modified Cas9 proteins lies in their ability to be fused with other functional domains, turning them into multifunctional tools. Base editing involves the fusion of nCas9 or dCas9 with deaminase enzymes that chemically convert one DNA base into another without introducing harmful double-strand breaks and thus can act independently of HDR mechanisms. For instance, cytosine base editors change a C-G base pair into a T-A pair, while adenine base editors convert an A-T base pair into G-C [55, 56]. Furthermore, RNA base editing with modified Cas13 represents a refined approach that delivers transient and reversible edits at the RNA level without altering the underlying DNA [57]. Both techniques enable precise, single-base modifications, making it especially effective for correcting or introducing point mutations linked to genetic diseases. However, the main limitations of base editors include unwanted edits within the editing window, indel formation, off-target RNA editing, and the larger size of the base editor complex compared to conventional Cas9, which complicates its delivery.

Prime editing is a more recent innovation that combines nCas9 with a reverse transcriptase enzyme. This allows for the introduction of specific changes, including small insertions, deletions, or base substitutions, directly at the target site, guided by a prime editing guide RNA (pegRNA). Unlike traditional CRISPR/Cas9 genome editing, prime editing directly incorporates new genetic information into the DNA, also resulting in higher precision and fewer unintended mutations [58]. However, the low efficiency and the ever-increasing complexes are the major limitations.

Beside editing/altering of DNA or RNA sequences, epigenome editing, CRISPR interference (CRISPRi), and CRISPR activation (CRISPRa) are further variations of the CRISPR/Cas9 system designed for precise control of gene expression [59–62]. In epigenome editing, dCas9 is fused to enzymes that modify the epigenetic landscape (DNA or histone modifications), influencing gene expression through changes in chromatin structure. For example, fusing dCas9 with methyltransferases can add methyl groups to cytosine bases, resulting in gene silencing, while attaching it to histone acetyltransferases or deacetylases can activate or repress gene expression through histone modification. In CRISPRi, dCas9 is typically fused to a repressive transcription factor or protein (such as KRAB-Krueppel-associated box), which blocks the transcription machinery or alters chromatin structure at the target locus, leading to gene silencing. On the contrary, CRISPRa results in an activation of gene expression. Here, dCas9 is linked to an activating transcription factor or complex (such as VP64 or p300) and directed to a gene’s promoter or enhancer region, where the activator domain recruits the transcription machinery to enhance gene expression. Recently, it has been shown that CRISPRa restores gene expression and corrects functional impairments associated with *TTN* truncation variants, the most common genetic alteration found in individuals with DCM [63]. CRISPRa has also been successfully demonstrated in vivo by targeting the *Mef2d* and *Klf15* loci, two well-characterised genes critical for cardiac hypertrophy and homeostasis. The described mouse model enables the enhancement of gene expression through the use of endogenous regulatory elements and demonstrates its potential for multiple applications in controlling transcription in cardiomyocytes of the postnatal heart [64]. Another study demonstrated that CRISPRi can effectively silence both the wildtype and mutant alleles of *CALM2*, successfully reversing calmodulinopathy – a life-threatening genetic arrhythmia syndrome – in hiPSC-CMs [65]. In addition, CRISPRi/a screens have been successfully used to identify therapeutic targets related to cardiac phenotypes [66]. A highly effective approach in functional genomics for studying genotype-phenotype correlations combines CRISPRi/a with the Perturb-seq method, which captures the full transcriptomic response to single-gene manipulation [67].

In recent years, genome editing has been increasingly explored for translational applications. Therapeutic genome editing holds great promise for the treatment of cardiogenetic diseases, as it enables targeted, durable and potentially curative interventions in living organisms. Recent studies have highlighted its significant potential in vivo, demonstrating substantial progress in this area [68–73]. Although challenges persist, particularly regarding the safe and efficient delivery of genome editing tools, ongoing research is steadily advancing the field, bringing it closer to clinical applications that could markedly enhance patient outcomes in cardiovascular medicine.

## Advances in cardiac tissue engineering

The pioneering work of Moscona et al. in 1959 showed that embryonic chick cardiomyocytes form spontaneously beating cardiospheres, which represents the basis for various cardiac tissue models [74]. To date, tremendous advancements have been made to generate complex disease modelling approaches intergating bioengineering technololgies. In addition, access to the different cardiovascular cell types derived from hiPSCs, as above mentioned, has further enabled the creation of more complex constructs to model cell-cell interaction and microenvironmental crosstalk within 3D cardiac tissues that more accurately mimic the heart’s architecture and function. In general, cardiac tissue engineering relies on three main strategies: (I) self-assembly of cells and extracellular matrix, (II) specialised devices or tools to direct cardiac tissue formation, and (III) 3D bioprinting for precise structural control (Fig. 1). Each strategy presents distinct strengths and limitations regarding scalability, complexity, and potential for clinical application, as discussed in the following sections.

(I) Cardiac organoids and microtissues

The lack of precise terminology often leads to confusion in the classification of 3D cardiac tissue structures; however, several key features help to distinguish between them. A key similarity between these two models is that neither requires an external extracellular matrix to initiate cellular self-assembly, indicating that they are capable of organising and developing independently to some degree. However, they differ significantly in their self-assembly dynamics and structural complexity. Both, organoids and microtissues, are commonly generated by promoting cellular aggregation in low-adhesion multi-well plates or bioreactors. Their formation requires relatively low cell numbers enabling the simultaneous production of hundreds to thousands of replicates.

Cardiac organoid is a widely used term for organised 3D structures that mimic the morphogenetic processes of in vivo heart development to some extent. These organoids, including multilineage organoids [75], gastruloids [76], heart-forming organoids [77, 78], and cardioids [79], can further be distinguished based on their complexity (cavitiy-forming) and the specific aspects of heart development they replicate. Recently, Schmidt et al. have successfully generated cardioids that represent all major embryonic heart compartments, which functionally integrate into multi-chamber structures with a shared lumen. This advanced model has been effectively used to study how genetic mutations, teratogens and drugs cause compartment-specific defects in the developing human heart in vitro [80].

In contrast, microtissues and assembloids are adaptable multicellular structures that self-assemble through the forced aggregation of terminally differentiated cells (either CMs only or in combination with non-CMs) [81]. Although these models may have limited cellular diversity, they provide a controlled microenvironment that is ideal for studying cell-cell interactions or to test functional integration of cell types in a simplified tissue environment. A three-cell type microtissue model was for example utilised to investigate arrhythmogenic cardiomyopathy (ACM), a genetic heart disease characterised by arrhythmias and fibrofatty deposits. Incorporating hiPSC-derived cardiac fibroblasts from ACM patients into these microtissues triggered arrhythmias, even in the presence of healthy cardiomyocytes and endothelial cells [82]. This disruption was linked to impaired communication between cardiomyocytes and fibroblasts, suggesting a key role in ACM-related arrhythmias [82]. Recently, assembloids combining atrial, atrioventricular canal, and ventricular spheroids have been developed to effectively simulate the atrioventricular conduction axis [83]. This advanced model identified intracellular calcium misregulation as the underlying cause of *LMNA*-associated atrioventricular block, providing a powerful tool for studying cardiac arrhythmias [83].

(II) Engineered heart tissues

Over the last two decades, a variety of engineered heart tissue (EHT) models have been developed. Generally, these models are constructed by integrating terminally differentiated cells – such as those derived from hiPSCs or primary cells, including cardiomyocytes, endothelial cells, and fibroblasts – with hydrogel scaffolds made from materials like collagen, fibrin, or Matrigel. Unlike cardiac organoids and microtissues, EHTs are shaped using molding technologies. In general, EHTs aim to replicate adult heart tissue structure and properties, rather than mimicking cardiac development, but they display limited self-organisation and patterning. Maturation is achieved using methods like mechanical stretching or electrical stimulation. One of the first experimental setups of EHTs was developed in 1997 by Eschenhagen et al. [84]. This approach involved embryonic chick cardiomyocytes combined with collagen, positioned between two Velcro-coated glass tubes, which allowed the constructs to anchor effectively and generate static forces during the tissue engineering process. Meanwhile, various types of EHTs have been developed, differing in their geometrical configurations. These include strip-shaped models [85–89], ring-shaped constructs [90, 91], patches [92], cardiac biowires [93, 94], spherical chambers [95], and tubular structures [96].

EHTs are highly valuable models for assessing contractile force and kinetics in cardiac disease modelling and drug testing [97]. However, their relatively labour-intensive manufacturing protocols, which require the use of specialised tissue-engineering equipment and, in many cases, large cell numbers, limit their broader application. [98]. To address these limitations, novel strategies, such as miniaturised microphysiological systems (MPS), are being developed. Benefiting from compact dimensions and the integration of microfluidic technologies, these systems improve nutrient supply, promote vascularisation and enhance perfusion. Moreover, MPS enable high-throughput drug screenings and can be tailored to study patient-specific disease mechanisms, making them valuable tools for personalised medicine [99].

(III) 3D bioprinted cardiac tissues

In recent years, 3D bioprinting has gained significant attention as an innovative technique for creating the next generation of advanced tissue models closely mimicking the structure and function of native cardiac tissue. These tissues hold great potential for applications in regenerative therapies, disease modelling, and drug testing, offering a more personalised and accurate approach to understanding heart disease and developing new treatments. 3D bioprinting has been successfully applied in the context of cardiovascular diseases in several impactful ways: For heart valve formation, collagen-based valves have been bioprinted to mimic the mechanical properties of natural heart valves, enabling them to open and close under pulsatile physiological pressure and flow – making them a promising option for valve replacement therapies [100]. Additionally, bioprinted endothelial cells have been incorporated into cardiomyocyte structures to form perfusable vascular networks, facilitating the development of functional cardiac patches with integrated blood vessel-like structures that enhance viability and function when implanted [101]. In regenerative heart repair, 3D bioprinting holds promise for repairing damaged heart tissue, offering potential applications in treating myocardial infarctions or congenital heart defects by replacing or regenerating damaged heart muscle [102].

The principles of 3D bioprinting build upon the traditional techniques of 3D printing technology. This process typically employs light-based or extrusion-based 3D printers enabling precise and automated control over material deposition [103, 104]. The bioink – a mixture of living cells (e.g. hiPSC-CMs only or combined with non-CMs) and hydrogels (natural or synthetic extracellular matrix) – is deposited layer by layer onto a designated surface, enabling the creation of complex, 3D tissue constructs [105]. This method provides an unprecedented level of control, allowing for the generation of engineered tissues with greater complexity [100]. It enables the precise modulation of various factors, including cell density, cellular heterogeneity, matrix stiffness, and construct size, which are critical for replicating the nuanced properties of native tissues. The challenge, however, lies in reducing/avoiding cell stress during the printing process, while also accurately reproducing the complex composition and architecture of the native cardiac extracellular matrix [106]. Continued advances in stem cell biology and 3D bioprinting technology will be critical to pushing the boundaries of biofabrication and enabling the generation of heart muscle tissue that incorporates the complex hierarchical structure, precise cell types, and extracellular matrix required to restore optimal contractile function.

## Conclusion

Cardiovascular diseases remain the leading cause of death globally, and existing preclinical models, including traditional animal models, often fall short in accurately replicating human cardiac physiology and drug responses. Over the past two decades, substantial progress has been made in leveraging hiPSC-based models to study heart diseases. However, with the increasing availability of hiPSC-derived 2D and 3D models and the advent of advanced genome editing technologies, selecting the most appropriate model has become more complex and now depends on specific research objectives. A key challenge in hiPSC-based cardiogenetic disease modelling lies in determining which structural and functional features need to be accurately mimicked. While simpler 2D models may be adequate for large-scale screenings, studies focusing on subtle phenotypic signatures may benefit from more sophisticated platforms that promote tissue maturity. The ultimate goal is to use disease models more strategically, facilitating a deeper and more precise understanding of the underlying pathomechanism. Integrating personalised heart models into drug development and early preclinical proof-of-concept studies holds great potential to enhance the precision and effectiveness of treatment strategies.
